# The Antifungal Peptide MCh-AMP1 Derived From *Matricaria chamomilla* Inhibits *Candida albicans* Growth via Inducing ROS Generation and Altering Fungal Cell Membrane Permeability

**DOI:** 10.3389/fmicb.2019.03150

**Published:** 2020-01-21

**Authors:** Sima Sadat Seyedjavadi, Soghra Khani, Ali Eslamifar, Soheila Ajdary, Mehdi Goudarzi, Raheleh Halabian, Reza Akbari, Hadi Zare-Zardini, Abbas Ali Imani Fooladi, Jafar Amani, Mehdi Razzaghi-Abyaneh

**Affiliations:** ^1^Department of Mycology, Pasteur Institute of Iran, Tehran, Iran; ^2^Department of Clinical Research, Pasteur Institute of Iran, Tehran, Iran; ^3^Department of Immunology, Pasteur Institute of Iran, Tehran, Iran; ^4^Department of Microbiology, School of Medicine, Shahid Beheshti University of Medical Sciences, Tehran, Iran; ^5^Applied Microbiology Research Center, Systems Biology and Poisonings Institute, Baqiyatallah University of Medical Sciences, Tehran, Iran; ^6^Department of Microbiology, Faculty of Medicine, Urmia University of Medical Sciences, Urmia, Iran; ^7^Hematology and Oncology Research Center, Shahid Sadoughi University of Medical Sciences, Yazd, Iran

**Keywords:** antifungal peptides, peptide synthesis, *Candida albicans*, antifungal activity, time killing assay, electron microscopy, mode of action, ROS generation

## Abstract

The rise of antifungal drug resistance in *Candida* species responsible for life threatening candidiasis is considered as an increasing challenge for the public health. MCh-AMP1 has previously been reported as a natural peptide from *Matricaria chamomilla* L. flowers with broad-spectrum antifungal activity against human pathogenic molds and yeasts. In the current study, the mode of action of synthetic MCh-AMP1 was investigated against *Candida albicans*, the major etiologic agent of life-threatening nosocomial candidiasis at cellular and molecular levels. *Candida albicans* ATCC 10231 was cultured in presence of various concentrations of MCh-AMP1 (16–64 μg/mL) and its mode of action was investigated using plasma membrane permeabilization assays, reactive oxygen species (ROS) induction, potassium ion leakage and ultrastructural analyses by electron microscopy. MCh-AMP1 showed fungicidal activity against *Candida albicans* at the concentrations of 32 and 64 μg/mL. The peptide increased fungal cell membrane permeability as evidenced by elevating of PI uptake and induced potassium leakage from the yeast cells. ROS production was induced by the peptide inside the fungal cells to a maximum of 64.8% at the concentration of 64 μg/mL. Scanning electron microscopy observations showed cell deformation as shrinkage and folding of treated yeast cells. Transmission electron microscopy showed detachment of plasma membrane from the cell wall, cell depletion and massive destruction of intracellular organelles and cell membrane of the fungal cells. Our results demonstrated that MCh-AMP1 caused *Candida albicans* cell death via increasing cell membrane permeability and inducing ROS production. Therefore, MCh-AMP1 could be considered as a promising therapeutic agent to combat *Candida albicans* infections.

## Introduction

*Candida albicans* is one of the most common nosocomial pathogens, which is responsible for various diseases that range from superficial to life-threatening systemic infections, especially in immunocompromised patients. Despite the increase in the incidence of candidiasis a limited number of anti-fungal drugs are available for the treatment of life-threatening fungal infections. Also, the increased resistance of *Candida* species to these conventional antifungal drugs has caused major concerns (Santos et al., 2018). As a result, there seems to be a necessity to develop new antifungal agents.

Both experimental and clinical studies in past decade have revealed that antimicrobial peptides (AMPs) are appropriate templates as a potential new class of therapeutics (Teixeira et al., 2012). Natural AMPs are identified as small (12–50 amino acids), cationic, amphipathic, and with variable lengths, which commonly isolated from animals, plants, bacteria, or fungi (Zasloff, 2002; Hilpert et al., 2005). They are also recognized as important components of the innate immune system against invasive pathogens (Li et al., 2012).

Despite extensive research on developing safe and novel AMPs, little has been documented about AMPs with antifungal activity probably due to the unexplored importance of fungal infections on human health (Marcos et al., 2012). Today, more than 3000 AMPs can be found in different AMP databases while, only a few peptide-based drugs, are available in the market and preclinical stage^1^ ^,2^ ^,3^. Unraveling the mechanism of action of AMPs facilitate the discovery and commercializing novel potent AMPs as next generation therapeutic agents (Mahlapuu et al., 2016).

The majority of AMPs function by increasing permeability and disrupting of microbial membranes, resulting in the leak of cellular contents and cell death (Huang et al., 2011; Teixeira et al., 2012; Lv et al., 2014). In addition the formation of reactive oxygen species (ROS) has been proposed to play a key role in the fungicidal mechanism of some antifungal peptides, such as, mellitin (Park and Lee, 2010), pleurocidin (Cho and Lee, 2011), and papillocin (Hwang et al., 2011). In the other hand, AMPs can inhibit the synthesis of intracellular molecules, such as DNA, RNA, and proteins (Brogden, 2005).

A large number of peptides isolated from different plant species appear to play an essential role in the protection of plant against pathogenic organisms. AMPs of a plant origin display broad-spectrum antimicrobial activities and seem to be capable of rapid killing. AMPs of plant origin are, therefore, promising natural antimicrobials for use in human healthcare as possible alternatives of chemically developed antimicrobial agents (Tam et al., 2015).

In our previous study, MCh-AMP1 (LSVKAFTGIQLRGVCGIEVKARG) (∼2402.8 Da), a novel antifungal peptide, was isolated from *Matricaria chamomilla* and found to be active against *Candida* species, while it did not show any obvious hemolytic effect on *in vitro* (Seyedjavadi et al., 2019). In the current study, mode of action of MCh-AMP1 was investigated against *C. albicans* with special focus on fungal cell integrity and permeability.

## Materials and Methods

### Chemicals and Reagents

2,7-dichlorofluorescein diacetate (DCFH-DA), amphotericin B, and propidium iodide (PI) were obtained commercially (Sigma-Aldrich, MO, United States). Sabouraud Dextrose Broth (SDB) and Sabouraud Dextrose Agar (SDA) were obtained from Merck (Darmstadt, Germany). All other reagents and chemicals were of analytical grade prepared from international companies.

### Fungal Strain and Growth Media

*Candida albicans* ATCC 10231 was maintained as frozen stock with glycerol at −80°C. Fresh cultures of the fungus prepared by sub-culturing on Sabouraud dextrose agar (SDA) at 28°C for 24 h were used throughout the study. In order to prepare cell suspension, one colony was picked from the SDA cultures and re-suspended in Sabouraud dextrose broth (SDB) to obtain a concentration of 1 × 10^6^ cells/mL.

### Peptide Synthesis and Characterization

MCh-AMP1 23-mer (LSVKAFTGIQLRGVCGIEVKARG) was custom-synthesized by Biomatik Co. (Ontario, Canada) with >95% purity (Supplementary Figure S1) according to standard solid phase methods (Merrifield, 1986).

### Antifungal Susceptibility Testing

Minimum inhibitory concentrations (MIC) for the peptide has been measured as described previously (Li et al., 2015; Seyedjavadi et al., 2019). Briefly, fungal cells, diluted to 5 × 10^5^ cells/mL in Sabouraud dextrose broth medium, incubated with several concentrations of the peptide at 35°C for 24 h. The MIC was taken as the lowest concentration that will prevent the growth of the fungal cells. In this study, amphotericin B, a conventional antifungal agent, was exploited as positive control. For minimal fungicidal concentration (MFC) determination, 50 μL cultures at concentration equal to or above MIC were plated on Sabouraud dextrose broth plate for CFU counting. After incubation at 35°C for 24 h, the MFC was defined as the lowest concentration killing at least 99.9% of the primary inoculums.

### Growth Inhibition Kinetics

Yeast growth inhibition assay was performed as previously described with slight modifications (Broekaert et al., 1990). Briefly, an overnight culture of *C. albicans* was adjusted to 10^6^ cells/mL in fresh SDB. After this, MCh-AMP1 was added in appropriate final concentrations (16, 32, and 64 μg/mL) and incubated at 28°C. Then; the growth kinetic was monitored at an optical density at 600 nm (OD_600_) for 8 h.

### Killing Kinetics

The time-killing kinetics study of MCh-AMP1 against cells was carried out according to standard microbiological techniques with few modifications (Mandala et al., 1998). Briefly, logarithmic cultures were diluted in fresh SDB to an inoculum size of 1 × 10^6^ cells/mL. Next, the cell suspension was incubated using MCh-AMP1 at appropriate final concentrations (16, 32, and 64 μg/mL). The solutions were incubated at 28°C via shaking at 120 rpm. Next, 30 μL of the sample was removed at specific time intervals (0, 1, 2, 3, 4, 5, 6, 7, 8 h), serially diluted in phosphate-buffered saline (PBS), pH 7.4, and plated on SDB in triplicate. After incubation at 28°C for 8 h, colony forming units (CFU) were counted. Amphotericin B was used as positive control. The results were represented as the average of triplicate measurements from three independent assays. The rate and extent of killing are also expressed as the mean log_10_ CFU/mL versus incubation time.

### Membrane Permeabilization Assay

#### Flow Cytometry Analysis

To determine the membrane permeability of MCh-AMP1, the propidium iodide (PI) uptake assay was used according to the flow cytometry method described by Wang et al., 2015 with minor modifications (Wang et al., 2015). *C. albicans* cells were grown and diluted to 1 × 10^6^ cells/mL, as previously described. Next, the cells were incubated with different concentrations of MCh-AMP1 (16, 32, and 64 μg/mL) and 2 μg/mL amphotericin B (positive control) for 3 h at 28°C via constant shaking (120 rpm). Subsequently, the filtered PI solution (50 μg/mL) was added, and further incubation was done for 15 min at 25°C in the dark place, followed by PBS washing. The percentage of PI positive cells was determined using fluorescence-activated cell sorting (FACS) Calibur flow cytometer (BD Biosciences, San Jose, CA, United States). The PI-treated and -untreated fungal cells were used as the negative control and blank, respectively. The data of three independent experiments are reported in this study.

#### Fluorescence Microscopy

*Candida albicans* cells (1 × 10^6^ cells/mL) were exposed to different concentrations of the peptide (16, 32, and 64 μg/mL) and 2 μg/mL amphotericin B (positive control) then incubated for 3 h at 28°C via constant shaking (120 rpm). The suspension was incubated with the PI solution (50 μg/mL) again for 15 min at 25°C in the dark, and then washed in PBS. The microscopic analysis was carried out using fluorescence microscopy (Eclipse 80i- Nikon-Japan), with appropriate filters (excitation/emission wavelength: 530/590 nm) (Wang et al., 2015). The untreated cells served as the negative control. The data of three independent experiments are reported in this study.

#### Potassium Release Assay

The potassium (K^+^) release assay was performed by measuring the extracellular K^+^ concentration released from *C. albicans* cell suspension, using a previously described method with slight modifications (Tao et al., 2014). Briefly, The *C. albicans* cells were harvested and resuspended with sterile deionized water then were exposed to MCh-AMP1 at different concentrations (16, 32, and 64 μg/mL) and 2 μg/mL amphotericin B (positive control) via constant shaking at 120 rpm at the given intervals (28°C). After centrifugation for 5 min, the percentage of K^+^ released into the supernatant was measured using flame atomic absorption spectroscopy. Cells that were only incubated with deionized water were used as the control. The data of three independent experiments are reported in this study.

### Measurement of Reactive Oxygen Species (ROS)

The intracellular ROS was measured using the fluorometric assay with 2′,7′-dichlorofluorescin diacetate (DCFH-DA) as previously described by Li et al. (2014) with slight modifications. Briefly, *C. albicans* cells (1 × 10^6^ cells/mL) were exposed to the different concentrations of peptide (16, 32, 64 μg/mL) and 2 μg/mL amphotericin B (positive control) for 3 h at 28°C via constant shaking (120 rpm). After incubation, 10 μM of DCFH-DA dye was added to the cell suspensions. The fluorescence intensity of ROS was measured in a FACS Calibur flow cytometer (BD Biosciences, San Jose, CA, United States). The untreated cells were used as the negative control. The data of three independent experiments are reported in this study.

### Electron Microscopy

To determine morphological changes of fungal cells in presence of MCh-AMP1 by scanning electron microscopy (SEM), method of Lyu et al. (2016) was used with slight modifications. A fungal suspension (1 × 10^6^ cells/mL) was exposed to 32 μg/mL of the peptide for 3 h and centrifuged for 10 min. Following incubation, the cells were centrifuged three times for 5 min. The pellet was then fixed with 2.5% (v/v) glutaraldehyde for 3 h at 4°C. After washing the pellet in 0.1% PBS three times, the samples were post-fixed in 1% osmium tetroxide in PBS for 1 h at room temperature. The fixed sample was washed with PBS, followed by dehydration using ethanol series (25, 50, 75, 95, and 100%) for 10 min and then absolute alcohol for 45 min. The samples were dried in critical–point with CO_2_, coated with a thin layer (20–30 nm) of gold-palladium, and observed under an analytical SEM microscope JEOL JSM-6510LA. The yeast cells, grown in the absence of the peptide, were run based on the same protocol as the control.

Transmission electron microscopy (TEM) was also performed, as described previously with some modifications (Bozzola and Russell, 1999). In brief, the fungal sample was initially prepared as described earlier for SEM analysis. After pre-fixing with 3% glutaraldehyde overnight at 4°C and washing the pellet with 0.1% PBS three times, the cell pellets were post-fixed with 1% osmium tetroxide in PBS for 70 min. Then, the samples were washed twice with PBS dehydrated in gradually increasing acetone solutions, and embedded in Epon 812. The ultrathin sections were cut using an ultra-microtome, stained with uranyl acetate and lead citrate, and finally observed using a Zeiss EM-900 TEM apparatus at 80 kV.

### Statistical Analysis

All the statistical analyses were performed using GraphPad Prism version 8.01 (GraphPad Software, San Diego, CA, United States) and analysis of variance followed by One way ANOVA. Group data were compared to untreated (Control) and group expressed as mean ± standard error. *p* ≤ 0.05 was considered as statistically significant. All tests were repeated in triplicate. ^∗^*p* ≤ 0.05, ^∗∗^*p* ≤ 0.01, ^∗∗∗^*p* ≤ 0.001.

## Results

### Peptide Characterization

Mass spectrometry analysis of the synthesized MCh-AMP1 confirmed the molecular weight of 2402.8 Da for the peptide (Supplementary Figure S1a). As shown in Supplementary Figure S1b in HPLC analysis, the peptide was found to be pure by a single sharp peak with the retention time (RT) of 8.947 min.

### Anticandidal Activity of MCh-AMP1

The minimum inhibitory and fungicidal concentrations (MIC and MFC) for MCh-AMP1 against *C*. *albicans* ATCC 10231 were 16 μg/mL and 32 μg/mL and both the MIC and MFC for amphotericin B were 2 μg/mL (Seyedjavadi et al., 2019).

### Growth Inhibition Kinetic Assay

**FIGURE 1:**
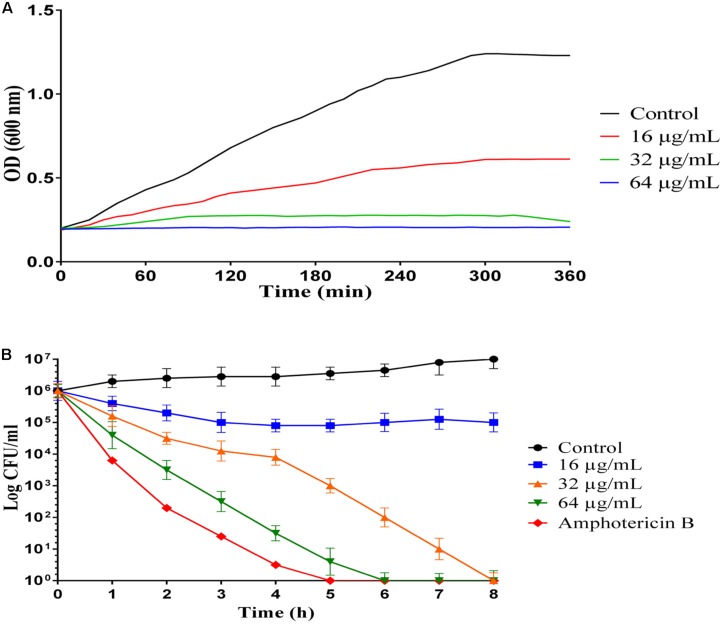
Inhibition and killing kinetics of MCh-AMP1 against *C. albicans*. **(A)** Optical density measurements at 600 nm of exponentially growing *C. albicans*, with and without peptide at 16–64 μg/mL concentrations. **(B)** Time-killing kinetics of MCh-AMP1 against *C. albicans* ATCC 10231. *C. albicans* cells (1 × 10^6^ cells/mL) suspensions were treated with different concentrations of MCh-AMP1 (16–64 μg/mL). Each data point represents mean ± SD of three separate experiments in triplicate sets each.

Growth inhibition kinetic of MCh-AMP1 were evaluated against *C. albicans* ATCC10231, using different concentrations of the peptide (16, 32, 64 μg/mL) at various intervals (Figure 1A). Growth inhibition results showed that the light absorbance rate at a wavelength of 600 nm (OD) in various concentration of MCh-AMP1 is decreased with increasing the concentration of the peptide. Optimal density for the control is increased during presumed time intervals. The OD rate of MCh-AMP1 in the low concentration (16 μg/mL) is slightly increased, while it is not changed at high concentrations of the peptide (32 and 64 μg/mL) during all time of incubation. Our data showed that the growth of *C. albicans* cells was inhibited by peptide at concentrations of 32 and 64 μg/mL during all tested times.

### Time Killing Assay

The killing kinetics of MCh-AMP1 were determined against *C. albicans* ATCC10231, using different concentrations of the peptide (16, 32, 64 μg/mL) at various intervals (Figure 1B). A concentration-dependent candidacidal activity of MCh-AMP1 was detected. As presented in Figure 1B at concentrations of 32 μg/mL and 64 μg/mL, the yeast cells were completely killed after 8 and 6 h of incubation, respectively. The killing kinetic experiment showed that at 64 μg/ml of the peptide, cells were 100% growth inhibited and the majority of cells were dead. At 32 μg/ml concentration, cells were almost completely growth inhibited and pretty much dead. At 16 μg/ml concentration, cells were initially growth inhibited, but started growing again after 20 min and albeit at a reduced growth rate, they enter stationary phase at the same time as the control. This means that they adapted to the stress conditions. Amphotericin B at concentration of 2 μg/mL demonstrated the killing kinetics, by eradicating the fungal cells within 5 h.

### Effects of the Peptide on Cell Membrane Permeability

**FIGURE 2:**
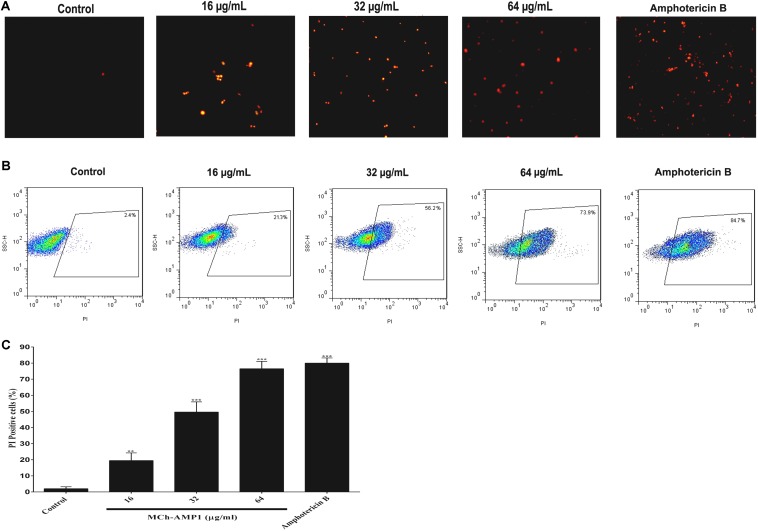
Effect of MCh-AMP1 on the cell membrane permeability of *C. albicans* ATCC 10231, measured by PI uptake. **(A)** Fluorescence microscopy analysis of membrane permeabilization assay by PI uptake. **(B)** Flow cytometric analysis of membrane permeabilization assay by PI uptake. Approximately 1 × 10^6^ cells/mL cells were incubated with MCh-AMP1 (16–64 μg/mL) and amphotericin B (2 μg/mL) for 3 h. **(C)** Histogram analysis shows the percentage of PI-positive *C. albicans* cells. Data are presented as the means ± SD of three independent experiments. The results are based on three independent experiments and represent the average, standard deviation, and *P*-values (^∗∗^*p* ≤ 0.01, ^∗∗∗^*p* ≤ 0.001) denote statistically significant differences from the effect of MCh-AMP1 on the control cells.

In this study, the effect of MCh-AMP1 on the fungal cell membrane integrity was investigated using the PI uptake procedure. PI could pass through only damaged membranes to bind with nucleic acids, and producing a red fluorescence under the fluorescence microscope. The results of PI staining of *C. albicans* cells at different concentrations of MCh-AMP1 for 3 h via fluorescence microscopy and flow cytometry are shown in Figure 2. As shown in Figure 2A under the view of fluorescence microscope the number of dead *C. albicans* cells (red) increased when the MCh-AMP1 concentration was increased from 16 to 64 μg/mL, however, there was no fluorescence in the control. Moreover, the flow cytometry assay was performed to verify the PI uptake. As depicted in Figure 2B, after treatment with 16 μg/mL of MCh-AMP1, 21.3% of cells showed PI fluorescent signals. Also, more than 56.2 and 73.8% of cells were labeled fluorescently after treatment with 32 μg/mL and 64 μg/mL of MCh-AMP1, respectively, while only 2.4% were labeled in non-exposed control fungal cells (Figure 2C). The increased membrane permeabilization is too slow to be direct permeabilization or membrane damage via the formation of pores, channels, carpeting or detergent action. This action is by an unknown mechanism which could entail membrane damage by ROS (discussed below). In the case of amphotericin B (2 μg/mL) 84.7% of cells were labeled fluorescently.

### Potassium Leakage

**FIGURE 3:**
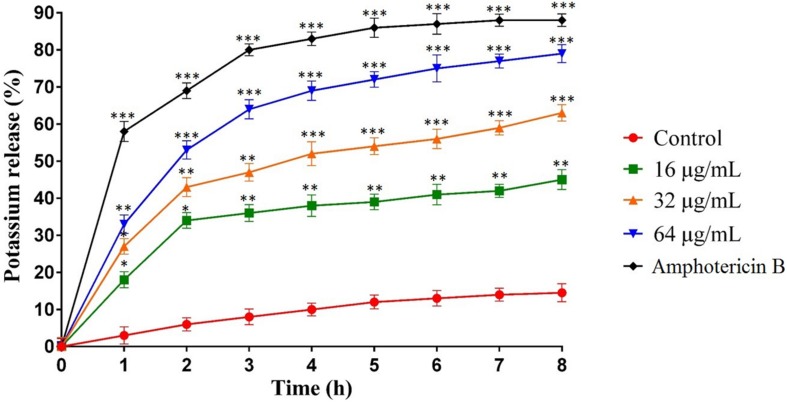
Effect of MCh-AMP1 on the potassium release of *C. albicans* ATCC 10231 is indicated. *C. albicans* cells were treated with different concentrations of MCh-AMP1 (16–64 μg/mL) and amphotericin B (2 μg/mL) for 3 h. The results are based on three independent experiments and represent the average, standard deviation, and *P*-values (^∗^*p* ≤ 0.05, ^∗∗^*p* ≤ 0.01, ^∗∗∗^*p* ≤ 0.001) denote statistically significant differences from the effect of MCh-AMP1 on the control cells.

The potassium leakage test was carried out to determine probable membrane permeability in *C. albicans* ATCC10231. Figure 3 shows the extracellular concentration of K^+^ in the *C. albicans* suspension that increased slowly over time in the control group, reaching 17% after 8 h. However, the release of K^+^ in *C. albicans* suspensions treated with MCh-AMP1 was higher at all intervals (Figure 3). At 16 μg/mL, MCh-AMP1 induced approximately 44% of K^+^ release after 8 h. The K^+^ release was increased gradually by increasing the concentrations of MCh-AMP1. The findings showed that approximately 63 and 79% of K^+^ release occurred after 8 h at 32 μg/mL and 64 μg/mL, respectively. In addition, amphotericin B (2 μg/mL) induced approximately 88% of K^+^ release after 8 h incubation.

### ROS Induction Assay

**FIGURE 4:**
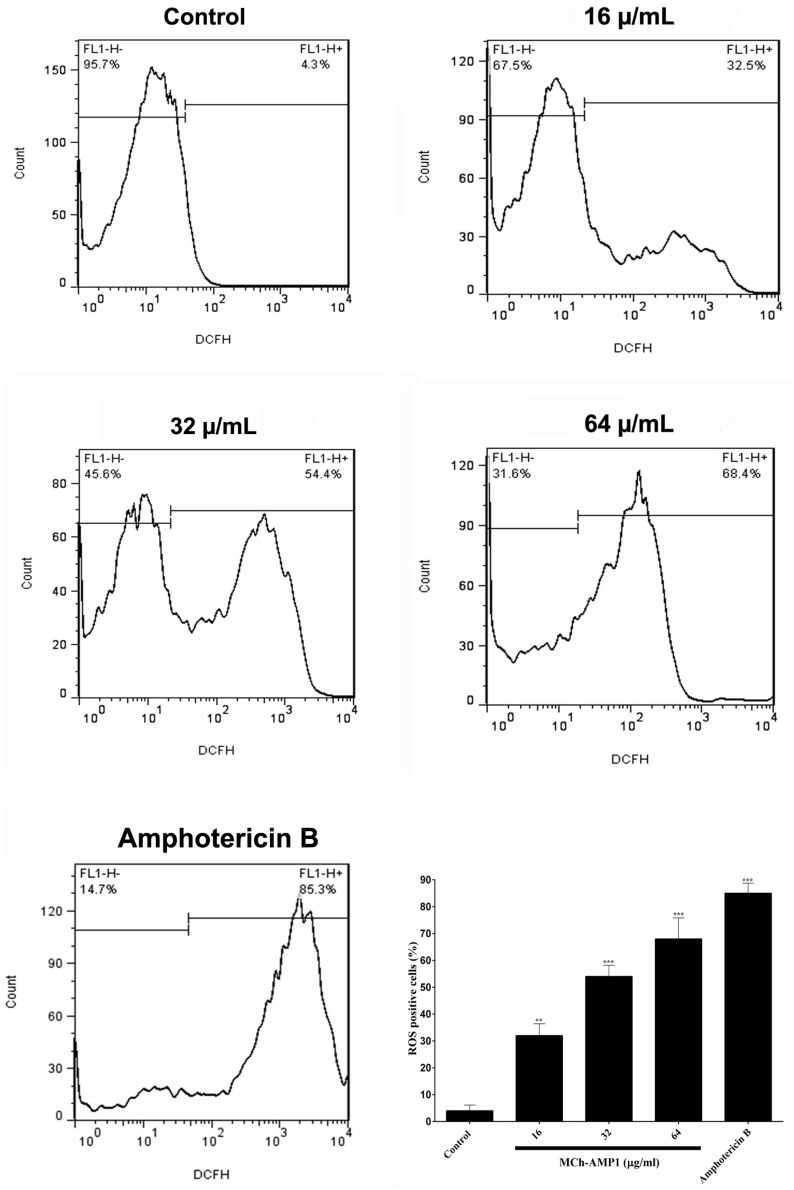
Effect of MCh-AMP1 on ROS generation of *C*. *albicans* ATCC 10231. Flow cytometric analysis of the intracellular ROS levels was analyzed using DCFH-DA. Cells were treated with different concentrations of MCh-AMP1 (16–64 μg/mL) and amphotericin B (2 μg/mL) for 3 h and stained with DCFH-DA. Histogram analysis shows the percentage of *C. albicans* cells that produce ROS. The results are based on three independent experiments and represent the average, standard deviation, and *P*-values (^∗∗^*p* ≤ 0.01, ^∗∗∗^*p* ≤ 0.001) denote statistically significant differences from the effect of MCh-AMP1 on the control cells.

The intracellular ROS production was monitored using a fluorescence dye, DCFH-DA in *C. albicans* cells after treatment with MCh-AMP1 at 16, 32, and 64 μg/mL for 3 h. Intracellular ROS is responsible for oxidizing DCFH-DA to DCF. As shown in Figure 4, a significant increase was observed in DCF fluorescence after 3 h of incubation with MCh-AMP1. The observed effect was dose-dependent, as 32% of the cells showed ROS-positive staining after treatment with 16 μg/mL of MCh-AMP1 and more than 54 and 68% showed ROS-positive staining after incubation with 32 and 64 μg/mL of MCh-AMP1, respectively (Figure 4). These results indicate that MCh-AMP1 produced intracellular ROS, leading to intracellular oxidative damage and possibly membrane damage. In addition, ROS production was observed, after incubation of *C. albicans* cells with 2 μg/mL amphotericin B.

### Electron Microscopy

**FIGURE 5:**
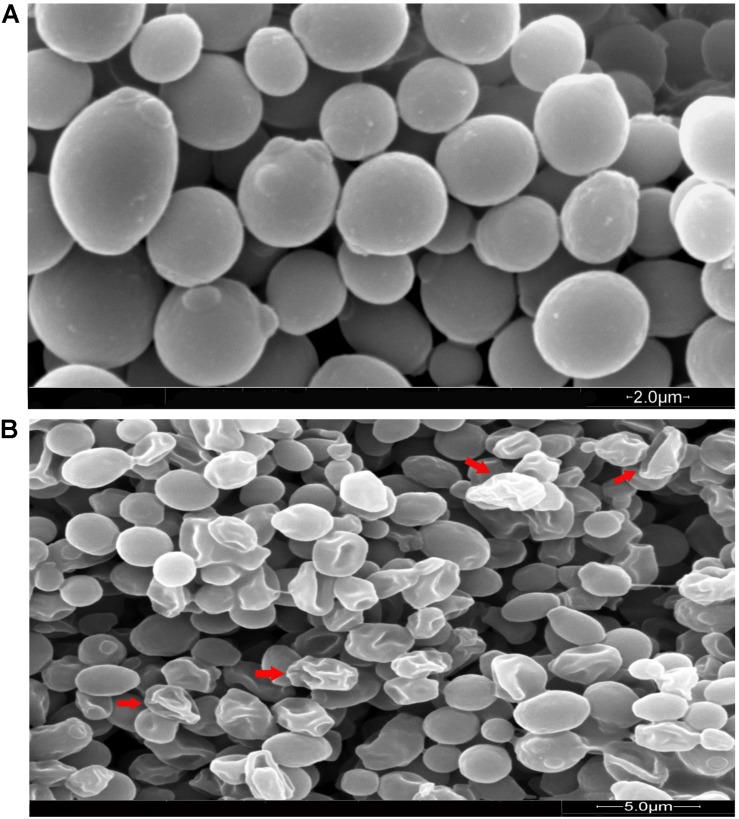
Scanning electron microscopy of *C. albicans* cells treated with MCh-AMP1. Approximately 1 × 10^6^ yeast cells were incubated without (**A**, control) or with MCh-AMP1 (32 μg/mL) **(B)** for 3 h. **(A)** SEM images of the untreated cells showed cells with normal shapes and smooth surfaces. **(B)** MCh-AMP1 treated cells showed a series of characteristic alterations, deep wrinkles and deformity (red arrows) along with oozing out of the intracellular content.

In order to investigate the effect of MCh-AMP1 on *C. albicans*, the morphology and ultrastructure of fungal cells were analyzed by scanning (SEM) and transmission (TEM) electron microscopy. SEM images of the untreated cells showed cells with normal shapes and smooth surfaces (Figure 5A), while MCh-AMP1 treated cells showed a series of characteristic alterations, deep wrinkles and deformity (red arrows) along with oozing out of the intracellular content (Figure 5B).

**FIGURE 6:**
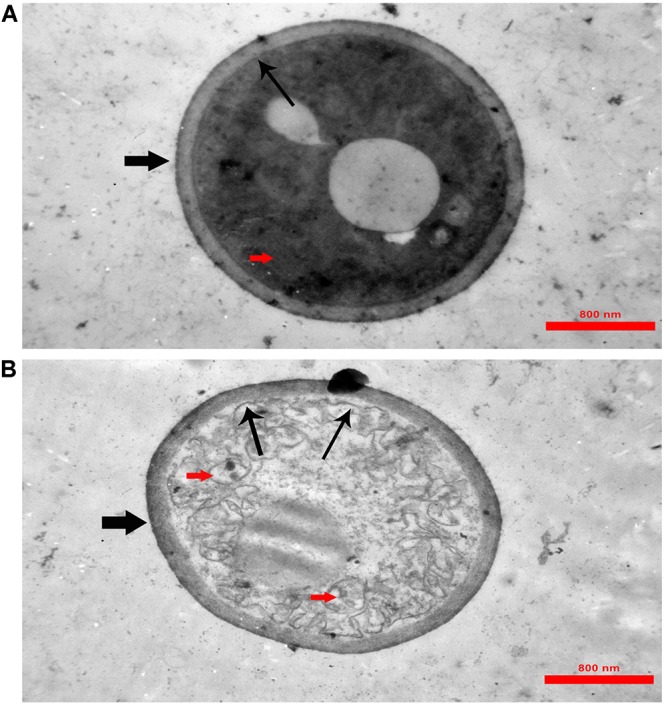
Transmission electron microscopy of *C. albicans* cells treated with MCh-AMP1. Approximately 1 × 10^6^ yeast cells were incubated without (**A**, control) or with MCh-AMP1 (32 μg/mL) **(B)** for 3 h. **(A)** Untreated *C. albicans* cells showed intact cell walls (black wide arrows), cell membranes (black narrow arrows) and organelles (red arrow). **(B)** Exposure to MCh-AMP1 resulted in morphological changes including loss of the structural integrity of the cell membrane and shrunk cytoplasm membrane detached from the cell wall (black narrow arrows), cytoplasmic depletion of fungal cells and destruction of intracellular organelles especially cell mitochondria (red arrows). The cell wall was not affected by the peptide (black wide arrow).

In TEM analysis, untreated *C. albicans* cells showed intact cell walls (black wide arrows), cell membranes (black narrow arrows) and organelles (red arrow) (Figure 6A). Exposure to MCh-AMP1 resulted in morphological changes including loss of the structural integrity of the cell membrane and shrunk cytoplasm membrane detached from the cell wall (black narrow arrows), cytoplasmic depletion of fungal cells and destruction of intracellular organelles especially cell mitochondria (red arrows) (Figure 6B). However, damage to the cell wall was not observed (black wide arrow).

## Discussion

The current study was conducted with the aim of understanding the mechanism of action of a novel AMP, MCh-AMP1, derived from *M. chamomilla*. To explore the probable mechanism of action of MCh-AMP1, we performed growth inhibition and killing kinetics studies. Based on our findings, MCh-AMP1 exhibited antifungal activities against *C. albicans* cells, and the concentration of MCh-AMP1 had a correlation with the antifungal activity. The concentration-dependent killing ability of various antifungal peptides such as polybia−CP and Protonectin against candida cells have been previously reported (Wang et al., 2015, 2016). The growth inhibition results for MCh-AMP1 corresponded to results of the time-kill kinetics. The growth of *C. albicans* cells is fully inhibited by MCh-AMP1 at the concentrations of 32 and 64 μg/mL during all time of incubation. The growth of *C. albicans* cells is repressed moderately at the concentration of 16 μg/mL. The time killing results showed that MCh-AMP1 could kill the *C. albicans* at the concentrations of 32 and 64 μg/mL within 8 and 6 h of incubation, respectively. The comparison of the results related to time-killing kinetic and growth inhibition kinetic showed that cells growth inhibition are occurred quickly after treatment by 32 μg/mL or higher doses of the peptide. While cells death is postponed up to 6–8 h at the similar doses. According to this observation, prolonged killing kinetics for AMPs that target intracellular compartments have been seen (Ganz, 2003). The antifungal mechanism of action of peptides with net positive charge is mostly associated with membrane permeabilization (Jenssen et al., 2006; van der Weerden et al., 2010). Since MCh-AMP1 is positively charged (+3), and contains many hydrophobic residues, it was concluded that the increased permeability of *C. albicans* cells membrane by MCh-AMP1 exposure might be due to this peptide’s interaction with the negatively charged components (such as phosphatidylserine and phosphatidylinositol) of the fungal surface and therefore disrupt the membrane barrier. Hence to determine whether the fungicidal effect observed from the killing kinetics experiments is associated to the ability of MCh-AMP1 to disrupt fungal membranes, we carried out PI uptake and potassium release assays. Our data from PI uptake assay showed that MCh-AMP1 can affect the integrity of fungal cell membrane and increase membrane permeabilization. In the majority of previous studies, *Candida* membrane damage has been reported after exposure to antifungal drugs (e.g., amphotericin B and fluconazole) (Ramani et al., 1997) and antifungal peptides (e.g., histatin 5, protonectin, and polybia−CP) (Helmerhorst et al., 1999; Wang et al., 2015, 2016). Our experimental findings of the PI uptake assay showed that at the concentration of 16 μg/mL approximately 20% of cells were PI-positive. However, based on the growth inhibition and killing kinetics studies, 60% growth inhibition was observed and 90% of cells were dead at this concentration and treatment time. At 32 μg/ml, about 55% of cells were PI positive but around 90–99% of cells were dead. These data together with the results of killing kinetics support the hypothesis that simple membrane disruption (i.e., pores or detergent-like mechanisms) is not the sole mechanism of action for MCh-AMP1. It seems that MCh-AMP1 could access to the cell membrane by crossing the cell wall during the yeast cells exponential growth phase, as it was shown that increased porosity allows the passage of compounds with low molecular weight from the fungal cell wall (Klis et al., 2014). Furthermore, any change in the permeability of the fungal cell membrane could results in the loss of the intracellular components, especially K^+^ ions. Our data from potassium release assay showed the limited potassium release at 16 and 32 μg/mL indicating that membrane disruption is not the sole mechanism of action of MCh-AMP1 and the opinion that the cell death induced by the peptide may occur via a different mechanism such as increasing intracellular ROS levels. In this context, our results indicated that MCh-AMP1 could induce the increase of ROS production in *C. albicans* cells. Different antifungal agents (For instance, amphotericin B and miconazole) (Delattin et al., 2014) and AMPs (For instance, histatin) (Giudici et al., 2006; Aerts et al., 2007; Mello et al., 2011) have shown to have ROS forming capacity. Like other antifungal peptides the mechanism of ROS production after treatment of *C. albicans* cells with MCh-AMP1 is unknown. Further studies will be required to clarify the mechanism of ROS production under the impact of this peptide. However, ROS are naturally generated during cellular metabolism and play a pivotal role in signaling and homeostasis. Various environmental stresses lead to enhanced production of ROS causing significant damage to cell structures. Moreover, excessive ROS damages a variety of molecules, including nucleic acids, proteins, and lipids that in addition to increasing cell membrane permeability can eventually cause cell death (Hwang et al., 2011).

In addition, SEM was employed to visualize any morphological changes induced by MCh-AMP1 in *C. albicans* cells. In the present study, the *C. albicans* cells treated with MCh-AMP1 show prominent shrinkage in the SEM that demonstrated the increased membrane permeability, and the leakage of ions or intracellular components. At the peptide concentration and time point used for SEM, fungal cells were mostly dead. However, around half of the cells do not show wrinkles and leakage. This is in accordance with the PI data of 50% PI uptake. This morphological effect has been previously reported for other antimicrobial peptides (Peng et al., 2010; Lee and Lee, 2015). Moreover, in the TEM analysis, we observed the detachment of plasma membrane from the cell wall, abnormal cell morphology, and destruction of cytoplasm and organelles without any obvious changes in the cell wall. According to the various deformities on *C. albicans* cells it seems that MCh-AMP1 achieves its anti-*Candida* activity by affecting the membrane permeability and various targets in the cells such as ROS. This is in accordance with the mode of antifungal action of polyenes such as nystatin and amphotericin B (Pierce et al., 2013) and antifungal peptides named RI18 (Lyu et al., 2016) and SKh-AMP1 (Khani et al., 2020). Existence of various targets in a microorganism cell for antimicrobial peptides, probably leads to decreasing the rapid development of microbial resistance (Maurya et al., 2011). However, it is hard to recognize the most important factors responsible for the candidacidal effects of MCh-AMP1, and it is technically challenging to characterize the processes leading to cell death. Overall, evidence suggests that all described mechanisms may play a critical role in the death of *C. albicans* cells.

## Conclusion

Results of the present study indicate that MCh-AMP1 acts as an antifungal natural compound via a combined mechanism of action involve increasing cell membrane permeability and inducing ROS production inside the *C. albicans* cells. Further investigations are needed to better understand about the exact intracellular targets of the peptide and how it causes the cell death. With respect to considerable antifungal activity of MCh-AMP1, it may be considered as a promising agent to use for therapeutic purposes against clinical life-threatening candidiasis.

## Data Availability Statement

The raw data supporting the conclusions of this article will be made available by the authors, without undue reservation, to any qualified researcher.

## Author Contributions

SS, JA, and MR-A conceived and designed the study. SS, SK, AE, SA, RH, HZ-Z, MG, AI, JA, and MR-A performed the experiments. SS, RA, JA, and MR-A analyzed and interpreted the data. SS, JA, and MR-A were drafted and written the manuscript. All authors approved the final version of manuscript. MR-A and JA supervised the study.

## Conflict of Interest

The authors declare that the research was conducted in the absence of any commercial or financial relationships that could be construed as a potential conflict of interest.

## Footnotes

1 https://asp.unmc.edu/AP/main.php

2 www.camp3.bicnirrh.res.in

3 https://dbaasp.org
