# Supplementary material for: The Antifungal Peptide MCh-AMP1 Derived From Matricaria chamomilla Inhibits Candida albicans Growth via Inducing ROS Generation and Altering Fungal Cell Membrane Permeability
Source: Front Microbiol. 2020 Jan 21;10:3150. doi: 10.3389/fmicb.2019.03150 (PMC6985553; doi:10.3389/fmicb.2019.03150)
Supplement: Supplementary file 1 [file Data_Sheet_1.DOC]

**Supplementary data**

**The antifungal peptide MCh-AMP1 derived from *Matricaria chamomilla* inhibits *Candida albicans* growth via inducing ROS generation and altering fungal cell membrane permeability**

Sima Sadat Seyedjavadi ^1^ , Soghra Khani ^1^ , Ali Eslamifar ^2^ , Soheila Ajdary ^3^ , Mehdi Goudarzi ^4^, [Raheleh Halabian](https://www.ncbi.nlm.nih.gov/pubmed/?term=Halabian R%5BAuthor%5D&cauthor=true&cauthor_uid=29372033) ^5^ Reza Akbari^6^ , Hadi Zare-Zardini ^7^ , Abbas Ali Imani Fooladi ^5^ , [Jafar Amani](https://www.ncbi.nlm.nih.gov/pubmed/?term=Amani J%5BAuthor%5D&cauthor=true&cauthor_uid=29372033) ^5**^ , Mehdi Razzaghi-Abyaneh ^1^*^*^*

^1^ *Department of Mycology, Pasteur Institute of Iran, Tehran, Iran*

^2^ *Department of Clinical Research, Pasteur Institute of Iran, Tehran, Iran*

^3^ *Department of Immunology, Pasteur Institute of Iran, Tehran, Iran*

^4^*Department of Microbiology, School of Medicine,*[*Shahid Beheshti University of Medical Sciences*](https://scholar.google.com/citations?view_op=view_org&hl=en&org=7030147968151678976)*, Tehran, Iran*

^5^ *Applied Microbiology Research Center, Systems Biology and Poisonings Institute, Baqiyatallah University of Medical Sciences, Tehran, Iran*

*^6^Department of Microbiology, Faculty of Medicine, Urmia University of Medical Sciences, Urmia, West Azerbaijan, Iran*

^7^*Hematology and Oncology Research Center, Shahid Sadoughi University of Medical Sciences, Yazd, Iran*

***Correspondence to:**

**Prof. Mehdi Razzaghi-Abyaneh**

Department of Mycology, Pasteur Institute of Iran, Tehran 1316943551, IRAN,

Tel: +98 21 64112804; Mobile: +98 912 3774027

E-mails: [mrab442@yahoo.com](https://mail.yahoo.com/neo/b/compose?to=mrab442@yahoo.com) & [mrab442@pasteur.ac.ir](https://mail.yahoo.com/neo/b/compose?to=mrab442@pasteur.ac.ir)

****Corresponding author**

**Dr. Jafar Amani**

Email: [jafar.amani@gmail.com](mailto:jafar.amani@gmail.com)

**
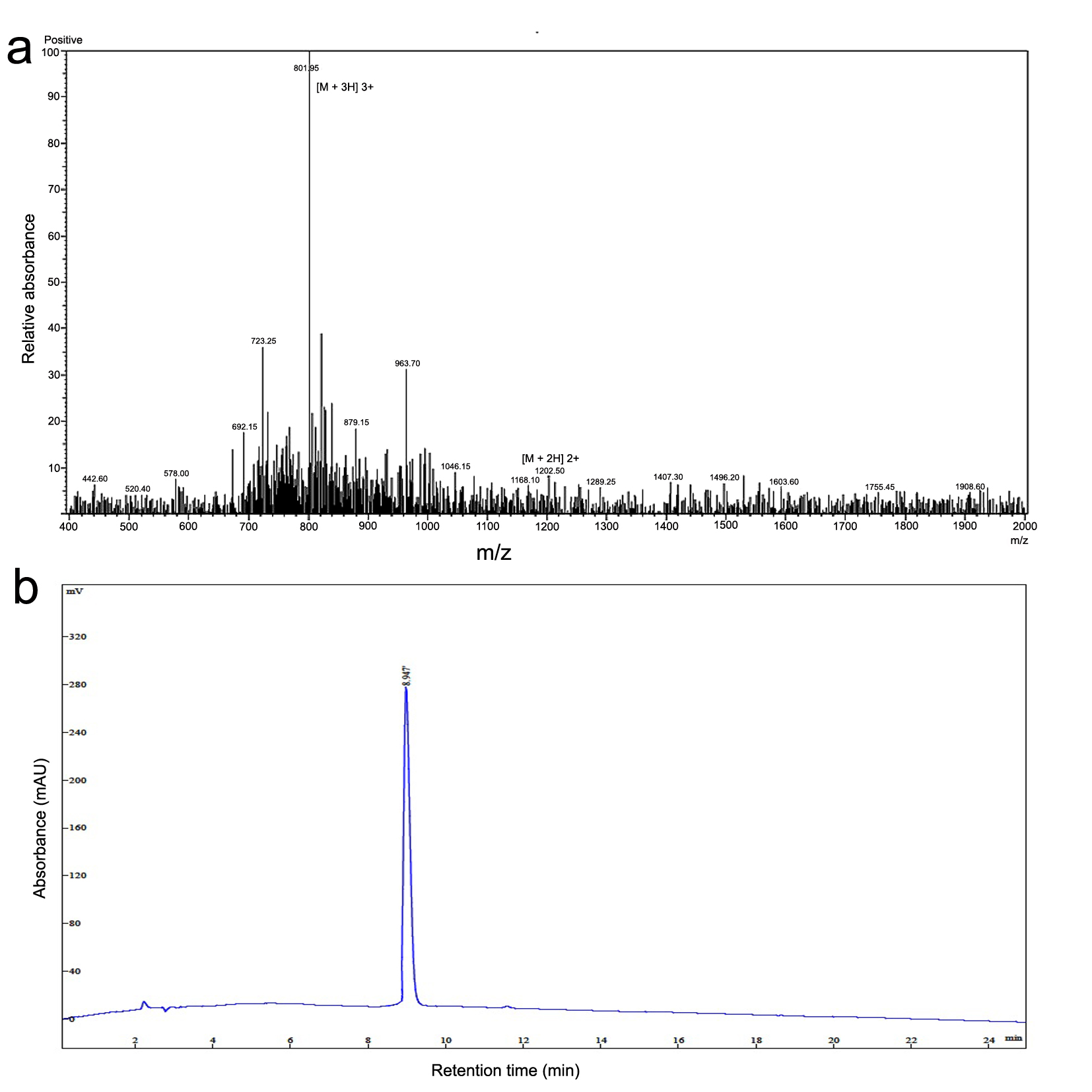
**

**Figure S1.** Mass spectrometry (a) and HPLC results (b) of synthesized MCh-AMP1 (LSVKAFTGIQLRGVCGIEVKARG) are shown. The peptide is shown as a single sharp peak in HPLC analysis. The purity of the synthetic peptide was confirmed using reverse-phase high-performance liquid chromatography (RP-HPLC >95% purity), followed by molecular weight determination via electrospray ionization-mass spectrometry (ESI-MS). HPLC system utilized a two-solvent gradient, with solvent A being composed of water with 0.1% TFA and solvent B composed of 99.9% acetonitrile with 0.1% TFA. A peptide sample was dissolved in 0.1% TFA in water and analyzed using a gradient of 0 to 75% solvent B over 30 minutes. The flow rate was set to 1.0 mL/min. and the detector monitoring the peptide bond absorbance at a wavelength of 220 nm.
